# Supplementary material for: Novel MAG Variant Causes Cerebellar Ataxia with Oculomotor Apraxia: Molecular Basis and Expanded Clinical Phenotype
Source: J Clin Med. 2020 Apr 23;9(4):1212. doi: 10.3390/jcm9041212 (PMC7230264; doi:10.3390/jcm9041212)
Supplement: Supplementary file 1 [file jcm-09-01212-s001.pdf]

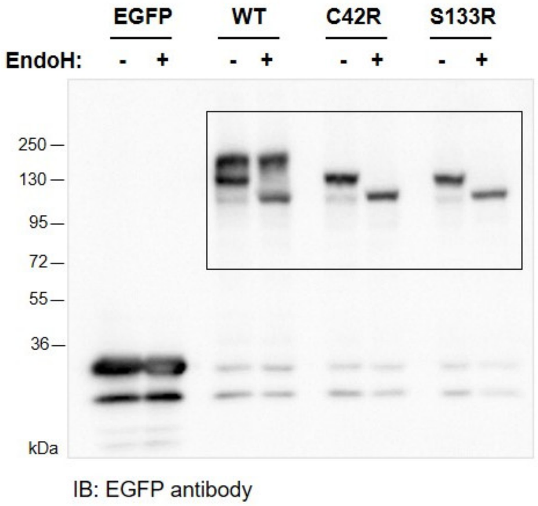

Figure S1. Original blot referring to main Figure 2B**.** Black box indicate the region selected for presentation in Figure 2B.

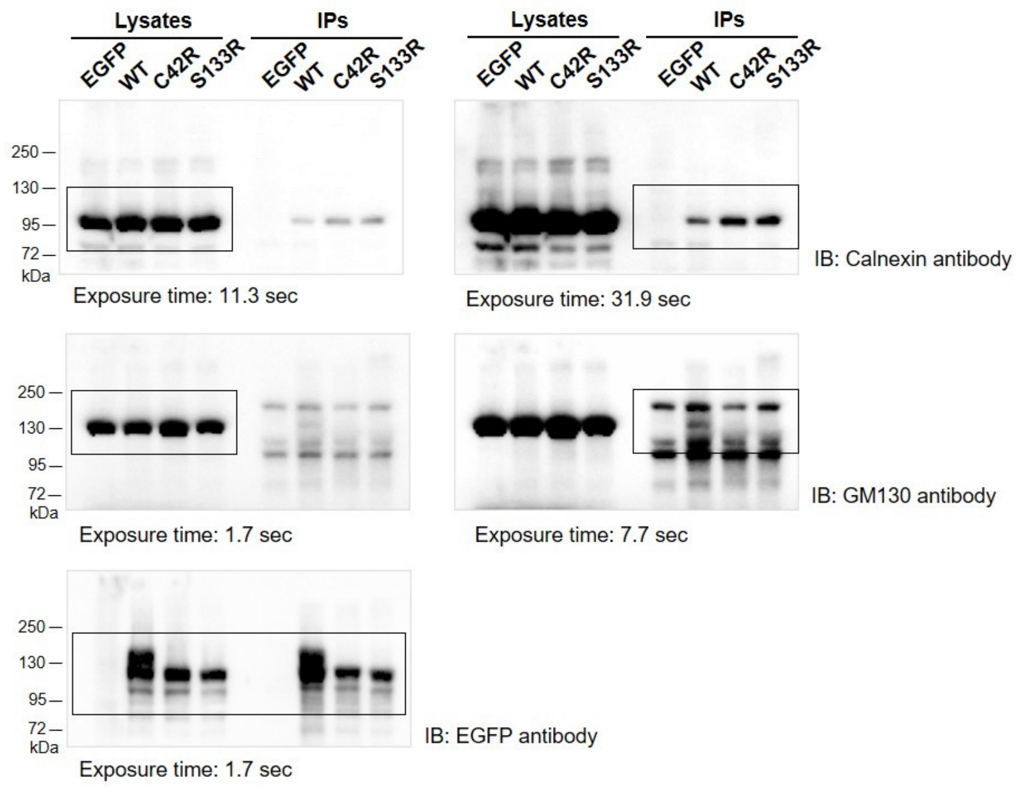

Figure S2. Original blots referring to main Figure 3B**.** Black boxes indicate the regions selected for presentation in Figure 3B.

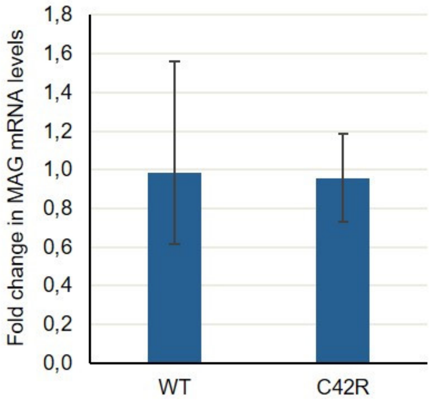

Figure S3. Quantitative analysis of *MAG* mRNA levels at 48h after transfection by real time-PCR. *MAG* levels were normalized towards *ACTB* levels. Data are presented the mean ± SD of three independent experiments.

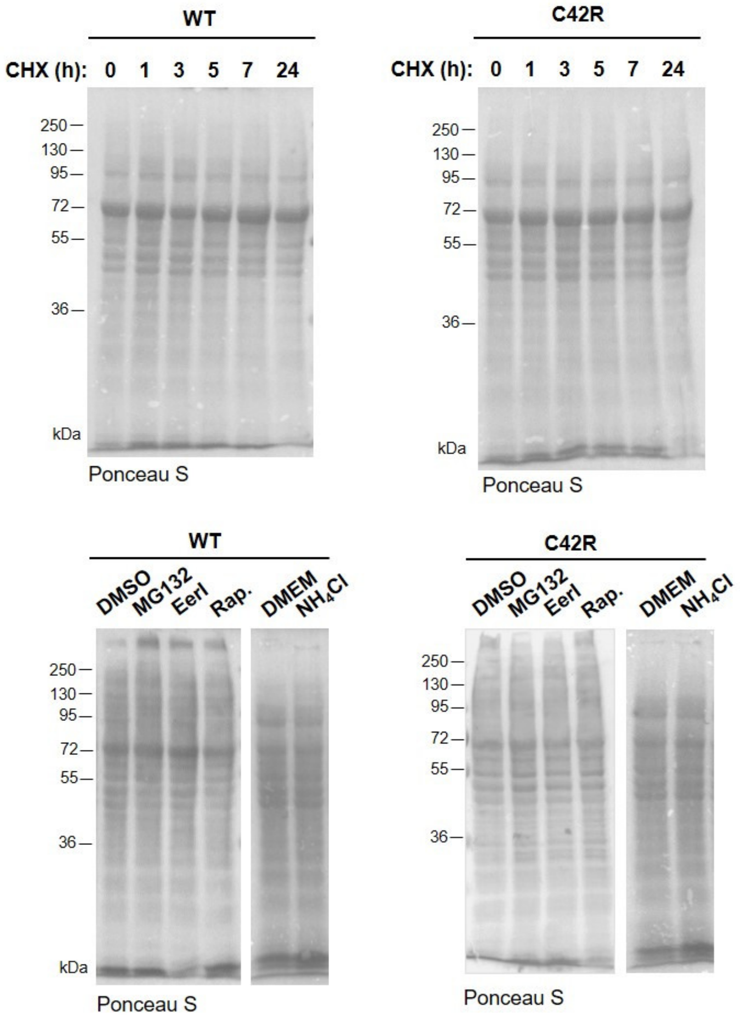

Figure S4. Original blots stained with Ponceau S referring to main Figure 4A and 4B**.**

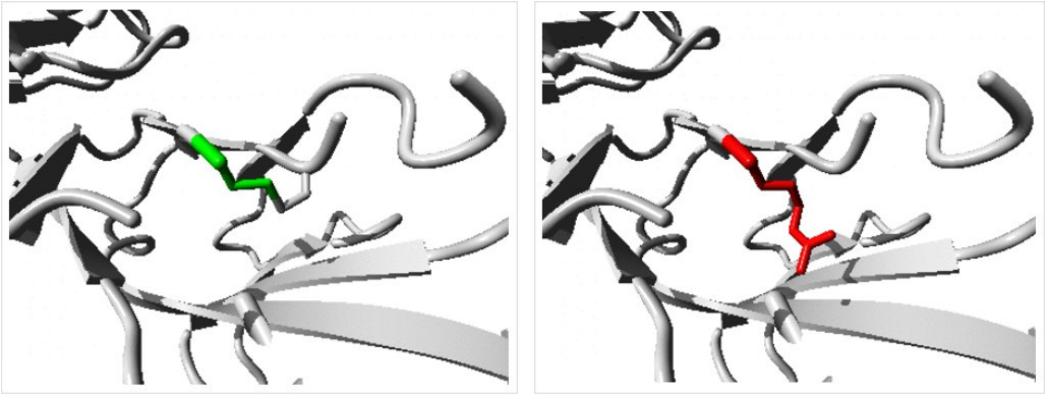

Figure S5. Visualization of the amino acids Cys42 (left figure, in green) and Arg52 (right figure, in red) on the protein structure of MAG, as seen on the HOPE report.

Table S1. Genome studio results showing loss of heterozygosity regions common to patients II.1 and II.3. The region where the *MAG* variant (c.124T>C) is located is highlighted.

Chromosome	Start	End	Size	Confidence score
3	75453100	107714075	32260975	8.983.827
4	52706270	60746052	8039782	7.159.385
6	57169715	58752089	1582374	3.857.165
6	61891638	63893492	2001854	9.347.172
7	118569946	119577292	1007346	259.741
9	4380096	9970747	5590651	1054.75
10	73917890	75407649	1489759	5.009.144
10	79729726	83594473	3864747	4.939.252
10	95543430	102996362	7452932	6.561.357
12	37927114	39229699	1302585	4.032.793
13	71081605	72411637	1330032	141.106
15	72097461	73320015	1222554	5.093.042
15	76397903	77825149	1427246	6.387.458
19	34142750	51480511	17337761	737.532
21	29803501	31111412	1307911	1.098.249

Table S2. List of homozygous variants located in loss of heterozygosity (LOH) regions. Intronic, UTR, intergenic and synonymous variants, and variants found in homozygosity in gnomAD were excluded.

Chr	Genomic position	RefSNPs	Gene	cDNA	Protein	Acession number	MAF	CADD_phred	SIFT score	Mutation Taster score	Polyphen2 score
**19**	35786593	-	*MAG*	c.124T>C	p.Cys42Arg	NM_002361.4	-	28	0	1	1
**19**	35617187	rs148987769	*LGI4*	c.1286T>C	p.Ile429Thr	NM_139284.3	0.2075%	31	0.06	0.986	0.992
**19**	39915627	rs200639701	*PLEKHG2*	c.3854A>G	p.Tyr1285Cys	NM_022835.3	0.003901%	26	0.13	1	0.99
**10**	96084656	rs771149406	*PLCE1*	c.6728G>A	p.Arg2243Gln	NM_016341.4	0.0008840%	28	0	0.999	1

The genomic position is relative to human assembly GRCh37 (hg19). Chr, chromosome; MAF, minor allele frequency (gnomAD, Non-Finish European).
